# Editorial: Perovskite materials for light-emitting devices

**DOI:** 10.3389/fchem.2022.1057245

**Published:** 2022-10-14

**Authors:** Ziming Chen, Tingting Shi, Xiaofang Jiang

**Affiliations:** ^1^ Department of Chemistry, Imperial College London, London, United Kingdom; ^2^ Guangdong Provincial Engineering Technology Research Center of Vacuum Coating Technologies and New Energy Materials, Department of Physics, Jinan University, Guangzhou, Guangdong, China; ^3^ Laboratory of Quantum Engineering and Quantum Material, School of Physics and Telecommunication Engineering, South China Normal University, Guangzhou, China

**Keywords:** perovskite emitters, perovskite light-emitting diodes, interfacial engineering, perovskite engineering, near-infrared emission

Over the past decade, great effort in the metal halide perovskite society has been devoted to the perovskite lighting field. The sky-rocketing external quantum efficiencies (EQEs) of perovskite lighting-emitting diodes (PeLEDs) from less than 1% to over 20% make perovskite emitters successfully join the solid-state lighting community (Chen et al., 2021a; Wu et al., 2021). The huge diversity of perovskite emitters with various compositions and dimensionality contributes to a colourful world of PeLEDs that their emission can be continuously tunned from violet to near-infrared, as well as white (*via* colour combination) (Chen et al., 2021a; Chen et al., 2021b).

The goal of this Research Topic is to show a wide range of possibilities of perovskite emitters for various functional light-emitting devices, to meet the requirement of potential applications in the future. Four original papers about the engineering of different types of perovskite emitters (including excitonic and non-excitonic systems), which are the core of PeLED devices, are collected here.

Excitonic perovskites, generally referred to low-dimensional perovskites such as two-dimensional (2D)/quasi-2D perovskites and perovskite quantum dots (QDs)/nanocrystals (NCs), are the most popular system and have established a large community in the perovskite light-emitting field, due to their outstanding optoelectronic properties like large exciton binding energy, low trap density, and high radiative efficiency. The tailoring of low-dimensional perovskites, *via* organic ligand engineering, perovskite compositional engineering, and controlling synthetic methods, is a powerful approach to modulating their nanostructure (e.g., multi-quantum wells and crystal sizes/shapes) as well as optoelectronic properties [e.g., emission colours and photoluminescence quantum yields (PLQYs)] (Dey et al., 2021; Zhang et al., 2021).

For instance, Wang et al. reported a simple strategy to improve the overall quality of quasi-2D perovskites. They found that in o-FPEA_2_Cs_*m*-1_Pb_*m*_Br_3*m*+1_ system (m ≥ 1, and o-F-PEABr = o-fluorophenylethylammonium bromide), the modulation of 2D perovskite layer thickness was important for the improvement of PLQY. A ∼60% PLQY was achieved in the *m* = 3 quasi-2D perovskite, while much lower PQLYs (<10%) in the *m* = 1 and 2 quasi-2D perovskites were obtained. This phenomenon was attributed to the strong electron-phonon coupling that caused non-radiative recombination (Chen et al., 2021a). Furthermore, using mixed ligands of o-F-PEABr and 2-aminoethanol hydrobromide (EOABr) to modulate the properties of *m* = 3 quasi-2D perovskite contributed to a ∼80% PLQY. The improvement from signal-ligand samples to dual-ligand samples, such as PLQYs (from ∼60% to 80%) and EQEs (from ∼5% to 10%), reflects that dual-ligand control is a feasible method to obtain high-quality perovskite emitters. However, more investigation is needed to fully understand the in-depth mechanism.

As another type of low-dimensional perovskite, perovskite QDs/NCs generally have sharp emissions and high colour purity. Interestingly, broad emissions are also achievable *via* ion doping, such as incorporating alkaline Earth metals or rare-earth ions into the perovskite lattice (Dey et al., 2021). For instance, Wang et al. successfully demonstrated broad-emission perovskite NCs by doping Mn^2+^ into CsPbCl_3_ (i.e., Mn:CsPbCl_3_), which generated a combined emission of a shape emission from CsPbCl_3_ (∼410 nm) and a broad emission from Mn^2+^ (∼600 nm). Moreover, they found that using 3-thienylboronic acid (TBA) to passivate the surface traps of Mn:CsPbCl_3_ NCs could suppress the non-radiative recombination, resulting in a huge improvement of PLQY from 46% (non-passivated one) to 93%. Another bonus for the TBA passivation was the significantly improved stability (including heating/moisture stability, storage stability, and purification stability) of the perovskite NCs. Both the enhanced PLQY and stability of Mn:CsPbCl_3_ NCs prove that surface passivation is an effective strategy to further strengthen the perovskite QD/NC quality.

Although PeLEDs based on excitonic perovskite systems operate efficiently at low charge-carrier density regions, they suffer from obvious efficiency droop at high charge-carrier density regions. In contrast, non-excitonic perovskite system like three-dimensional (3D) perovskites works reversely that better device performance can be achieved when charge-carrier density is high, because the radiative recombination rate (or bimolecular recombination rate) is proportional to the charge-carrier density in 3D perovskites (Xing et al., 2017). Therefore, 3D perovskites are proper choices for high-brightness PeLEDs which need to be injected with a large amount of current. For instance, Yuan et al. have demonstrated high-brightness green PeLEDs with a maximum luminance of 72,082 cd/m^2^, based on CsPbBr_3_-Cs_4_PbBr_6_ hybrid perovskites. They found that the excessive CsBr on the surface of the perovskite film could react with partial 3D CsPbBr_3_ to form Cs_4_PbBr_6_, which could modify the film morphology and passivate the crystal surface. More importantly, they successfully demonstrated a hole-transport-layer-free device based on a unique device architecture of ITO/Perovskite/LiF (8 nm)/Bphen (60 nm)/LiF (1 nm)/Al, and with which a device half-lifetime of 1,000 min was achieved. This report reflects that composition engineering is an effective strategy to optimise the 3D perovskite crystals and films for better light emission.

Regarding near-infrared emission, in terms of emission wavelength, 3D perovskites are also better candidates than low dimensional perovskites that tend to have bluer emission. The substitution of Pb^2+^ with Sn^2+^ in the perovskite lattice can lead to redshifts of emission to the near-infrared region (∼1,000 nm) (Liu et al., 2019). Moreover, the choice of A-site cations for these Sn/Pb mixed perovskites is also critical for their emission properties. Liu et al. has investigated how A-site cations of MA^+^, FA^+^, and Cs^+^ affected the quality of perovskites of ASn_x_Pb_1-x_I_3_ (x = 0, 0.2, 0.4, 0.6, 0.8, 1). They found that MASn_x_Pb_1-x_I_3_, FASn_x_Pb_1-x_I_3_, and CsSn_x_Pb_1-x_I_3_ had bandgaps ranging from 1.2 to 1.55 eV, 1.2–1.5 eV, and 1.3–1.7 eV, corresponding to near-infrared emission of 766–980 nm, 800–965 nm, 716–960 nm, respectively. However, only MASn_x_Pb_1-x_I_3_ and FASn_x_Pb_1-x_I_3_ perovskites were considered promising for lighting applications as CsSn_x_Pb_1-x_I_3_ perovskites showed weak emission, poor stability, and poor film morphology, which might relate to their small tolerance factor of the lattice (∼0.81).

In conclusion, in this Research Topic, we show three types of perovskite emitters (i.e., 0D perovskite QDs/NCs, 2D/quasi-2D perovskites, and 3D perovskites) with various optoelectronic properties for different lighting applications. We believe these reports could provide the perovskite lighting field with more inspiration and guidelines for the future development of perovskite light-emitting devices.
